# Food groups and micronutrients associated with chemotherapy-induced peripheral neuropathy in survivors of cancer post-neurotoxic treatment

**DOI:** 10.1007/s00520-025-09827-6

**Published:** 2025-08-23

**Authors:** Karly Miller, Suzanna Zick, Robert Ploutz-Snyder, Edward A. Ruiz-Narváez, Robert Knoerl

**Affiliations:** 1 School of Public Health, University of Michigan, 1415 Washington Heights, Ann Arbor, MI 48109 USA; 2 Department of Family Medicine, School of Public Health, Michigan Medicine, and Nutritional Sciences, Ann Arbor, MI 48105 USA; 3 University of Michigan School of Nursing, 400 North Ingalls St, Ann Arbor, MI 48109 USA

**Keywords:** Chemotherapy-induced peripheral neuropathy, Dietary intake, Food groups, Surveys and questionnaires

## Abstract

**Purpose:**

Determine the association of food group and micronutrient intake with chemotherapy-induced peripheral neuropathy (CIPN) among survivors of cancer.

**Methods:**

In this cross-sectional study, participants completed the PRO-CTACE™ numbness and tingling severity item and VioScreen™ Research Food Frequency Questionnaire to determine CIPN status and dietary intake, respectively. Separate covariate-adjusted linear, logistic, and ordered logistic regression models were used to calculate the mean food group and micronutrient amounts and the associations with CIPN status and severity. As a secondary analysis of prior research that was not powered to detect these associations, we considered *p* ≤ 0.10 as an indicator of relevance for future study.

**Results:**

A total of 136 participants completed the surveys and questionnaires. Participants with greater daily intake of refined grains (OR = 2.05; 95% CI, 1.22, 3.46) and less intake of tomatoes (*OR* = 0.10; 95% CI, 0.10, 1.14), fish (OR = 0.21; 95% CI, 0.06, 0.75), eggs (OR = 0.06; 95% CI, 0.01, 0.34), and selenium (OR = 0.96; 95% CI, 0.92, 1.00) were associated with increased odds of having CIPN. The odds of experiencing worse CIPN severity increased with each additional serving of refined grains (OR = 1.66; 95% CI, 1.11, 2.48) and decreased with each extra serving of poultry (OR = 0.58; 95% CI, 0.31, 1.08), fish (OR = 0.18; 95% CI, 0.05, 0.61), egg (OR = 0.08; 95% CI, 0.02, 0.39), legumes (OR = 0.04; 95% CI, 0.00, 1.49), and selenium (OR = 0.96; 95% CI, 0.93, 1.35).

**Conclusion:**

There are meaningful intake differences between participants with and without CIPN. Further research is needed to establish a dietary intervention using the findings in a larger population.

## Introduction

Chemotherapy-induced peripheral neuropathy (CIPN) is a side effect experienced by nearly 60% of survivors of cancer after receiving neurotoxic chemotherapy (i.e., taxane and platinum-based) [1]. Chemotherapeutic agents can cause damage to peripheral nerve structures (e.g., microtubules and ion channels) leading to CIPN, a debilitating condition presenting symptoms of numbness, tingling, and pain in the hands and feet [1, 2]. With the cancer survivor population continuing to grow with a projected increase of 24% in the next 8 years, CIPN incidence is expected to grow at a similar rate [3]. Despite moderate effectiveness, medicinal treatments are the primary approach to reducing CIPN symptoms [4, 5]. Duloxetine is the first-line treatment for the management of CIPN pain [6], but duloxetine is infrequently prescribed in practice (e.g., clinicians are unfamiliar or possible drug-drug interactions) [7, 8]. Among non-pharmacological options, the Society for Integrative Oncology recommends managing CIPN using integrative treatments such as acupuncture, reflexology, or acupressure [9]. However, the evidence to support the use of such integrative treatments is limited, reflecting the need for conclusive research resulting in more effective approaches.

Diet is a modifiable behavior that greatly impacts neural development and regeneration [10]. Dietary consumption may influence the incidence and severity of CIPN, as macronutrient and micronutrient components play significant roles in energy status and metabolism. High-quality nutrient intake provides enzymes and cofactors necessary to maintain a healthy peripheral nervous system (PNS) [10, 11]. Previous studies have supported the role of dietary nutrients on nerve function, suggesting their potential as a non-pharmacological approach to address CIPN in survivors of cancer post-treatment [5, 10–13]. Only a few studies have observed the relationships between diet quality and CIPN. Knoerl et al. (2024) conducted a cross-sectional study to explore the association between diet quality and CIPN severity among post-treatment cancer survivors who had received platinum or taxane-based chemotherapy. Results revealed that participants without CIPN self-reported better diet quality than those with CIPN. Further findings for unadjusted analyses demonstrated that cancer survivors without CIPN had higher consumption of fats and lower consumption of carbohydrates and added sugars than cancer survivors with CIPN [14]. However, less was known about specific food groups and micronutrients that were associated with less CIPN, which could be recommended by clinicians for patients with CIPN to inform future dietary interventions.

The primary aim of this secondary analysis was to determine the association among food group consumption, micronutrients, and CIPN status and severity among cancer survivors who had received platinum or taxane-based chemotherapy.

## Methods

### Design, sample, and setting

The data for this secondary analysis were from a cross-sectional study that aimed to determine the association between diet quality, macronutrients, and CIPN among post-treatment cancer survivors [14]. Briefly, the sample included 134 cancer survivors who were 3 months post-platinum or taxane-based chemotherapy treatment and did not report neuropathy due to other causes. Participants were categorized as experiencing CIPN (≥ 1/4) or not experiencing CIPN (score = 0) based on the Patient-Reported Outcomes Version of the Common Terminology Criteria for Adverse Events (PRO-CTCAE™) numbness and tingling severity item, which measures the severity of CIPN-related pain. Participants were recruited from the University of Michigan Rogel Cancer Center and nationwide via a social media campaign. The University of Michigan IRBMED (HUM00219474) reviewed and monitored the study. Participants completed a written informed consent via an electronic platform (i.e., SignNow).

### Measures and data collection

#### Data collection

Enrolled participants completed the VioScreen™ Research Food Frequency Questionnaire, PRO-CTCAE™ numbness and tingling items [15–17], U.S. Household Food Security Survey Module: Six-Item Food Security Module [18], demographic questions, and Godin and Shephard Leisure-Time Exercise Questionnaire [19–21] at one time point. The measures are described in more detail.

#### CIPN status and severity

The National Cancer Institute’s PRO-CTCAE™ numbness and tingling severity item measures CIPN severity from the previous 7 days in a numerical range from 0 to 4. Scores of 0 represent no CIPN, while scores from 1 to 4 indicate less to more severe levels of CIPN [22–24]. Previous studies exhibit the PRO-CTCAE™ numbness and tingling severity item as a reliable and validated measure to determine participants’ CIPN-related severity in a population of survivors of cancer [23, 24].

#### Food group and micronutrient intake

Eligible participants completed the VioScreen™ Research (VioCare) Food Frequency Questionnaire (FFQ), an online self-administered survey reflecting average dietary intake from the previous three months. The Nutrition Data System for Research software version 51 was developed at the University of Minnesota, Minneapolis, MN, by the Nutrition Coordinating Center to collect data that encompasses dietary patterns, intake estimates, and dietary analyses [25, 26]. VioScreen™ collects data from 156 food items and groups, further calculating caloric intake and percentage to determine the macronutrient and micronutrient composition of daily/weekly average food consumption utilizing visual examples of portion sizes [27]. A study evaluating the test–retest reliability of web-based FFQs compared to paper FFQs calculated the Pearson Correlation for macronutrient (*median* = 0.60–0.90) and micronutrient (*median* = 0.49–0.82) consumption [28]. Participants with caloric intake data < 500 kcals/day and > 5000 kcals/day were removed from the dataset as a control for quality.

VioScreen™ calculated dietary intake in servings per day or week for each food group and micronutrient. Food groups were determined using the 2015–2020 Dietary Guidelines for Americans (DGAs) United States Department of Agriculture (USDA) MyPlate (i.e., a visual guide to assist in making healthier food choices) recommendations [29]. The five food groups on the USDA’s MyPlate are fruits, vegetables, grains, protein, and dairy. Each food group is divided into additional subgroups specific to nutrient characteristics (e.g., dark green vegetables, orange and red vegetables, tomatoes, other vegetables, whole grains, refined grains, fruit, red meat, poultry, fish, eggs, nuts and seeds, and legumes) [29]. Micronutrients such as fat-soluble vitamins: vitamins A, D, E, and K; B vitamins: thiamin, riboflavin, niacin, pantothenic acid, pyridoxine, folate, and cobalamin; vitamin C; magnesium; selenium; calcium; iron; copper; and zinc were chosen based on prior research demonstrating an impact on peripheral nerve functionality and regeneration [5, 11–13].

#### Physical activity

The Godin and Shephard Leisure-Time Exercise Questionnaire is supported by evidence that indicates its validity for use in cancer survivor populations [19–21, 30]. It quantifies participant data regarding mild, moderate, and strenuous leisure-time physical activity. The questionnaire calculates weekly leisure activity scores by multiplying strenuous, moderate, and mild exercise frequency by 9, 5, and 3, respectively. Individuals who scored 24 or more units were interpreted as active, 14–23 units as moderately active, and 14 or less as insufficiently active/sedentary [21].

#### Covariate measures

Demographic information (i.e., age, sex, and race) was self-reported by all participants. BMI (kg/m^2^) was calculated using weight and height collected from previous cancer treatment appointments or self-reported if treatment was not from the Rogel Cancer Center. Average daily caloric intake for the previous 3 months was collected using data from the VioScreen™ FFQ. Food security status from the last 12 months was determined using the U.S. Household Food Security Survey Module: Six-Item Food Security Module [18]. A score was calculated using a range from 0 to 6, 0 to 1 representing high or marginal food security, 2 to 4 low food security, and 5 to 6 very low food security.

### Statistical analysis

Stata (v. 18) software was used for all statistical analyses [31]. We chose a level of significance at *p* ≤ 0.10 as this was a preliminary study and we view these results as hypothesis-generating [32]. Demographic and clinical characteristics of the participants were reported as *means* and *standard deviations *(*SD*) for continuous numerical variables and *n *(*%*) for categorical variables.

CIPN severity using the PRO-CTCAE™ numbness and tingling severity item was measured as a range of four increasing CIPN severity levels: 0, none; 1, mild; 2, moderate; 3, severe; 4, very severe. CIPN status was reported as a binary categorical variable using the results from the PRO-CTCAE™ numbness and tingling severity item (i.e., no CIPN or CIPN present). Participants reporting a CIPN severity level of 0 were placed in the “no CIPN” group, while participants reporting levels from 1 to 4 were placed in the “CIPN present” group. We adjusted for the following covariates: age, sex, race, body mass index (BMI), caloric intake, food security, and physical activity (e.g., a categorical value). Linear regression compared the mean intake of food groups and micronutrients with covariates between participants with and without CIPN. Ordinal regression compared the mean intakes among individuals with varying levels of CIPN severity. Logistic regression and ordered logistic regression were used to evaluate the odds between food group or micronutrient intake and CIPN status or severity, respectively. Servings of food groups and micronutrients were adjusted by average daily caloric intake among participants and multiplied by 1000 cal per day. Covariates were utilized in three separate models: Model 1 controlled for average daily caloric intake only; Model 2 controlled for average daily caloric intake, age, sex, race, BMI, and food security; Model 3 controlled for the same covariates as Model 2 with the addition of physical activity.

## Results

### Sample flow and demographics

Data for 136 participants were available for analysis from the primary study. In brief, the sample population was majority women (*n* = 127, 93%), White (*n* = 127, 93%), food secure (*n* = 131, 96%), and receiving paclitaxel chemotherapy (*n* = 93, 68%). The mean age of the participants was 54.45 (± 12.06) years with an average BMI of 27.98 (± 6.59) kg/m^2^ (Table 1). Data regarding physical activity was missing from 12 participants, reducing the sample size from *N* = 136 to *N* = 124 in Model 3.

**Table 1:** Demographic and clinical characteristics of the sample by case and control status of informing comparisons

Characteristic	CIPN present (*n* = 76)	No CIPN (*n* = 60)	Total (*N* = 136)	*p*-value^1^
Age at baseline*				**< 0.01**
*Mean (SD)*	58.55 (11.56)	49.25 (10.67)	54.45 (12.06)	
Biological sex				0.30
Female	69 (91%)	58 (97%)	127 (93%)	
Male	7 (9%)	2 (3%)	9 (7%)	
Race				0.22
Asian	2 (3%)	4 (7%)	6 (4%)	
Black or African American	0	1 (2%)	1 (1%)	
White	73 (96%)	54 (90%)	127 (93%)	
Unknown or do not wish to report	1 (1%)	0	1 (1%)	
Other	0	1 (2%)	1 (1%)	
Ethnicity				0.17
Hispanic or Latino	1 (1%)	0	1 (1%)	
Not Hispanic or Latino	74 (98%)	56 (93%)	130 (95%)	
Unknown or do not wish to report	1 (1%)	4 (7%)	5 (4%)	
Education				0.26
Some college or technical training	17 (22%)	7 (12%)	24 (18%)	
University undergraduate degree	22 (29%)	21 (35%)	43 (32%)	
University postgraduate degree	37 (49%)	32 (53%)	69 (51%)	
Marital status				0.24
Single	4 (5%)	4 (7%)	8 (6%)	
Married/partnered	52 (68%)	48 (8%)	100 (74%)	
Separated	1 (1%)	0	1 (1%)	
Divorced	13 (17%)	6 (10%)	19 (14%)	
Widowed	6 (8%)	1 (2%)	7 (5%)	
Missing	0	1 (2%)	1 (1%)	
Employment status*				**0.01**
Working full-time	29 (38%)	37 (62%)	66 (49%)	
Working part-time	5 (7%)	4 (7%)	9 (7%)	
Working at home	2 (3%)	3 (5%)	5 (4%)	
Working, but on medical leave	2 (3%)	1 (2%)	3 (2%)	
Student	0	1 (2%)	1 (1%)	
Retired	32 (42%)	9 (15%)	41 (30%)	
Not working	6 (8%)	5 (8%)	11 (8%)	
Godin and Shephard leisure-time exercise questionnaire^1^				0.27
Sedentary	12 (16%)	11 (18%)	23 (17%)	
Moderately active	10 (13%)	12 (20%)	22 (16%)	
Active	50 (66%)	29 (48%)	79 (58%)	
Missing	4 (5%)	8 (13%)	12 (9%)	
Food security				0.52
0–1	71 (93%)	60 (100%)	131 (96%)	
2–4	1 (1%)	0	1 (1%)	
5–6	4 (5%)	0	4 (3%)	
Cancer type				0.44
Gastrointestinal^a^	14 (18%)	5 (8%)	19 (14%)	
Breast	42 (55%)	45 (75%)	86 (63%)	
Gynecological^b^	14 (18%)	9 (15%)	23 (17%)	
Other^c^	3 (4%)	0	3 (2%)	
Multiple	2 (3%)	0	2 (1%)	
Missing	1 (1%)	1 (2%)	2 (1%)	
Cancer stage				0.15
Stage I	16 (21%)	21 (35%)	37 (27%)	
Stage II	22 (29%)	12 (20%)	34 (25%)	
Stage III	24 (32%)	15 (25%)	39 (29%)	
Metastatic	11 (14%)	12 (20%)	23 (17%)	
Unknown	3 (4%)	0	3 (2%)	
Treatment type^d^				
Paclitaxel*	44 (58%)	49 (82%)	93 (68%)	**< 0.01**
Docetaxel*	20 (26%)	7 (12%)	27 (20%)	**0.05**
Oxaliplatin*	16 (21%)	5 (8%)	21 (15%)	**0.06**
Carboplatin*	30 (39%)	14 (23%)	44 (32%)	**0.06**
Cisplatin	4 (5%)	0	4 (3%)	0.13
Years from last neurotoxic chemotherapy^1^*				**0.09**
* Mean *(*SD*)	3.44 (3.83)	4.39 (2.63)	3.86 (3.38)	
BMI (kg/m^2^)				0.26
* Mean *(*SD*)	28.54 (7.25)	27.27 (5.62)	27.98 (6.59)	
Caloric intake (kilocalories)^1^*				**0.05**
* Mean *(*SD*)	1835.50 (790.35)	1594.26 (618.48)	1729.07 (727.09)	
PRO-CTCAE severity*				** < 0.01**
0	0	60 (100%)	60 (44%)	
1	30 (39%)	0	30 (22%)	
2	35 (46%)	0	35 (26%)	
3	11 (14%)	0	11 (8%)	
4	0	0	0	
PRO-CTCAE interference*				**< 0.01**
0	26 (34%)	60 (100%)	86 (63%)	
1	28 (37%)	0	28 (21%)	
2	16 (21%)	0	16 (12%)	
3	6 (8%)	0	6 (4%)	
4	0	0	0	

Notes: Models 1 and 2 are presented; not all percentages sum to 100% due to rounding; categorical variables compared using exact tests; continuous variables compared using ttests; ^1^unequal variance adjustment; *statistically significant (*p*-value ≤ 0.10); ^a^gastrointestinal cancers include anal, colon, pancreatic, and rectal cancer types; ^b^gynecological cancers include ovarian, endometrial, fallopian tube, and uterine cancer types; ^c^other cancers include bladder, lung, and prostate cancer types; ^d^percentages do not sum to 100% due to participants receiving more than one type of neurotoxic agent

Bolded *p*-values indicate characteristics significantly associated with CIPN (*p* < 0.10)

### Food group and micronutrient mean daily intakes predicting the odds of CIPN status

Table 2 describes the covariate-adjusted mean daily food group and micronutrient consumption between groups with and without CIPN along with the odds of having CIPN. Results from the linear regression analyses controlling for caloric intake, age, race, sex, BMI, and food security (Model 2) revealed participants with CIPN consumed, on average, more refined grains (*p* = 0.01) and thiamine (*p* = 0.09) along with less eggs (*p* = 0.04), legumes (*p* = 0.05), and selenium (*p* = 0.02) compared to those without CIPN. Logistic regression analysis results reflect every serving increase of refined grains was associated with twice the odds of having CIPN (OR = 2.05; *p* = < 0.01; 95% CI, 1.22, 3.46). Other food groups and micronutrients such as tomatoes (OR = 0.10; *p* = 0.06; 95% CI, 0.10, 1.14), fish (OR = 0.21; *p* = 0.02; 95% CI, 0.06, 0.75), eggs (OR = 0.06; *p* = < 0.01; 95% CI, 0.01, 0.34), and selenium (OR = 0.96; *p* = 0.03; 95% CI, 0.92, 1.00) were associated with decreased odds of reporting CIPN with each additional serving.

Table

**Table 2:** Average adjusted food group and micronutrient intakes for survivors of cancer post-neurotoxic chemotherapy predicting the odds of chemotherapy-induced peripheral neuropathy (CIPN) adjusting for caloric intake, age, race, sex, BMI, and food security per 1,000 kilocalories

Dietary Component (*N*=136)	Average amount of intake by CIPN status		Mean adjusted difference *p-*value	OR (95% CI)^b^	OR *p*-value
	CIPN Present^a^ (*n*=76)	No CIPN^a^ (*n*=60)		Odds of having CIPN	
Food Groups					
Dark Green Vegetables (cups/day)	0.19 (0.14, 0.24)	0.19 (0.13, 0.25)	0.98	1.53 (0.44, 5.32)	0.50
Red and Orange Vegetables (cups/day)	0.07 (0.04, 0.09)	0.08 (0.04, 0.11)	0.77	3.11 (0.35, 27.68)	0.31
Tomatoes⏶ (cups/day)	0.18 (0.15, 0.21)	0.21 (0.17, 0.26)	0.18	0.10 (0.01, 1.14)	**0.06**
Other Vegetables (cups/day)	0.24 (0.20, 0.29)	0.26 (0.21, 0.31)	0.65	0.67 (0.14, 3.23)	0.62
Whole Grains (oz/day)	0.61 (0.46, 0.75)	0.57 (0.41, 0.72)	0.73	0.83 (0.44, 1.56)	0.55
Refined Grains*⏶ (oz/day)	1.68 (1.48, 1.88)	1.32 (1.14, 1.49)	**0.01**	2.05 (1.22, 3.46)	**<0.01**
Fruit (cups/day)	0.76 (0.61, 0.90)	0.80 (0.62, 0.97)	0.74	0.90 (0.52, 1.55)	0.71
Red Meat (oz/day)	0.17 (0.12, 0.22)	0.15 (0.10, 0.20)	0.70	0.83 (0.14, 4.76)	0.83
Poultry (oz/day)	0.41 (0.32, 0.51)	0.54 (0.39, 0.69)	0.17	0.66 (0.32, 1.34)	0.25
Fish⏶ (oz/day)	0.15 (0.09, 0.21)	0.22 (0.12, 0.31)	0.30	0.21 (0.06, 0.75)	**0.02**
Egg*⏶ (oz/day)	0.16 (0.12, 0.21)	0.26 (0.18, 0.33)	**0.04**	0.06 (0.01, 0.34)	**<0.01**
Nuts/Seeds (oz/day)	0.52 (0.35, 0.68)	0.39 (0.25, 0.54)	0.30	1.14 (0.85, 1.52)	0.40
Dairy (cups/day)	0.76 (0.63, 0.89)	0.68 (0.55, 0.80)	0.39	1.49 (0.63, 3.53)	0.37
Legumes* (cups/day)	0.05 (0.03, 0.06)	0.09 (0.05, 0.12)	**0.05**	0.18 (0.00, 16.74)	0.46
Micronutrients					
Vitamin A (IU/day)	6702.07 (5609.32, 7794.82)	5414.70 (4405.73, 6423.67)	0.11	1.00 (1.00, 1.00)	0.11
Vitamin D (IU/day)	122.61 (103.78, 141.44)	135.91 (112.05, 159.77)	0.42	1.00 (0.99, 1.00)	0.24
Vitamin E (IU/day)	10.50 (9.29, 11.71)	10.59 (9.20, 11.98)	0.93	1.01 (0.96, 1.07)	0.71
Vitamin K (mcg/day)	108.34 (89.09, 127.60)	94.84 (75.58, 114.11)	0.37	1.00 (1.00, 1.01)	0.11
Thiamin* (mg/day)	0.81 (0.77, 0.86)	0.75 (0.70, 0.80)	**0.09**	3.47 (0.42, 28.82)	0.25
Riboflavin (mg/day)	1.11 (1.03, 1.18)	1.15 (1.07, 1.24)	0.45	0.83 (0.33, 2.06)	0.68
Niacin (mg/day)	12.74 (11.78, 13.71)	13.28 (12.13, 14.43)	0.51	1.00 (0.95, 1.04)	0.85
Pantothenic Acid (mg/day)	3.58 (3.21, 3.95)	3.73 (3.29, 4.17)	0.62	1.01 (0.94, 1.09)	0.77
Vitamin B6 (mg/day)	1.15 (1.05, 1.25)	1.20 (1.09, 1.32)	0.53	0.94 (0.61, 1.44)	0.78
Folate (mcg/day)	151.82 (138.99, 164.65)	155.28 (140.28, 170.28)	0.75	1.00 (0.99. 1.01)	0.82
Vitamin B12 (mcg/day)	2.42 (2.13, 2.71)	2.60 (2.24, 2.96)	0.47	0.96 (0.84, 1.11)	0.61
Vitamin C (mg/day)	83.52 (71.40, 95.64)	74.91 (62.48, 87.33)	0.36	1.00 (1.00, 1.01)	0.30
Magnesium (mg/day)	197.57 (184.97, 210.17)	192.22 (178.21, 206.23)	0.60	1.00 (1.00, 1.01)	0.33
Selenium*⏶ (mcg/day)	55.58 (53.29, 57.86)	60.13 (57.31, 62.95)	**0.02**	0.96 (0.92, 1.00)	**0.03**
Iron (mg/day)	7.78 (7.25, 8.31)	7.30 (6.72, 7.87)	0.26	1.08 (0.91, 1.28)	0.38
Copper (mg/day)	0.85 (0.80, 0.91)	0.82 (0.76, 0.89)	0.57	1.67 (0.30, 9.19)	0.56
Zinc (mg/day)	5.97 (5.64, 6.30)	6.16 (5.77, 6.54)	0.50	0.89 (0.70, 1.13)	0.33
Calcium (mg/day)	582.53 (531.05, 634.01)	555.10 (499.04, 611.17)	0.51	1.00 (1.00, 1.00)	0.28

### Food group and micronutrient mean daily intakes predicting the odds of CIPN severity

Table 3 describes the mean daily food group and micronutrient intakes adjusted for caloric intake, sex, race, BMI, and food security between PRO-CTCAE severity levels (0–4) and the odds of experiencing greater CIPN severity. Ordinal linear regression results demonstrate participants with worse levels of CIPN severity consumed greater average intakes of refined grains (*p* = 0.09) and fewer nuts/seeds (*p* = < 0.01), legumes (*p* = 0.07), and selenium (*p* = 0.08). Ordered logistic regression results show a one serving increase of refined grains is associated with 1.66 times greater odds of increasing CIPN severity (OR = 1.66; *p* = 0.01; 95% CI, 1.11, 2.48). Conversely, a one serving increase in poultry (OR = 0.58; *p* = 0.08; 95% CI, 0.31, 1.08), fish (OR = 0.18; *p* = < 0.01; 95% CI, 0.05, 0.61), egg (OR = 0.08; *p* = < 0.01; 95% CI, 0.02, 0.39), legumes (OR = 0.04; *p* = 0.07; 95% CI, 0.00, 1.49), and selenium (OR = 0.96; *p* = 0.01; 95% CI, 0.93, 1.35) are associated with decreased odds of reporting worse CIPN.

**Table 3:** Average adjusted food group and micronutrient intake for survivors of cancer post-neurotoxic chemotherapy with ranging severity of chemotherapy-induced peripheral neuropathy (CIPN) adjusting for caloric intake, age, race, sex, BMI, and food security per 1000 kilocalories

Dietary component (*N* = 136)	CIPN severity^a^				Omnibus differences, *p*-value	OR (95% CI)^b^	OR *p*-value
	Average amount of intake by CIPN severity level						
	0	1	2	3		Odds of a greater CIPN severity	
	None(***n*** = 60)	Mild(***n*** = 30)	Moderate (***n*** = 35)	Severe (***n*** = 11)			
Food groups							
Dark green vegetables (cups/day)	0.20 (0.13, 0.26)	0.24 (0.14, 0.34)	0.15 (0.09, 0.21)	0.19 (0.05, 0.34)	0.50	1.19 (0.47, 3.01)	0.72
Orange vegetables (cups/day)	0.08 (0.04, 0.11)	0.08 (0.03, 0.13)	0.06 (0.03, 0.09)	0.07 (0.00, 0.13)	0.89	2.66 (0.39, 17.96)	0.31
Tomatoes (cups/day)	0.22 (0.17, 0.26)	0.19 (0.14, 0.24)	0.16 (0.12, 0.20)	0.18 (0.10, 0.26)	0.44	0.14 (0.01, 1.84)	0.13
Other vegetables (cups/day)	0.27 (0.21, 0.32)	0.30 (0.22, 0.38)	0.20 (0.15, 0.26)	0.22 (0.11, 0.32)	0.26	0.41 (0.11, 1.53)	0.19
Whole rains (oz/day)	0.56 (0.40, 0.73)	0.59 (0.37, 0.82)	0.63 (0.40, 0.86)	0.57 (0.20, 0.95)	0.98	0.99 (0.50, 1.98)	0.98
Refined grains*⏶ (oz/day)	1.31 (1.14, 1.49)	1.68 (1.39, 1.98)	1.64 (1.36, 1.93)	1.82 (1.26, 2.37)	**0.09**	1.66 (1.11, 2.48)	**0.01**
Fruit (cups/day)	0.81 (0.63, 0.98)	0.85 (0.61, 1.09)	0.73 (0.53, 0.94)	0.57 (0.28, 0.85)	0.57	0.73 (0.45, 1.20)	0.22
Red meat (oz/day)	0.15 (0.10, 0.20)	0.15 (0.08, 0.22)	0.16 (0.09, 0.23)	0.26 (0.05, 0.46)	0.70	1.58 (0.35, 7.04)	0.55
Poultry⏶ (oz/day)	0.55 (0.40, 0.70)	0.44 (0.28, 0.60)	0.42 (0.27, 0.56)	0.33 (0.12, 0.54)	0.48	0.58 (0.31, 1.08)	**0.08**
Fish⏶ (oz/day)	0.22 (0.12, 0.32)	0.19 (0.08, 0.31)	0.13 (0.05, 0.21)	0.13 (0.05, 0.21)	0.56	0.18 (0.05, 0.61)	**< 0.01**
Egg⏶ (oz/day)	0.26 (0.18, 0.34)	0.19 (0.11, 0.27)	0.14 (0.08, 0.20)	0.16 (0.05, 0.27)	0.16	0.08 (0.02, 0.39)	**< 0.01**
Nuts/seeds* (oz/day)	0.42 (0.27, 0.57)	0.79 (0.42, 1.16)	0.47 (0.25, 0.68)	0.16 (0.03, 0.30)	** < 0.01**	0.97 (0.82, 1.15)	0.71
Dairy (cups/day)	0.67 (0.54, 0.80)	0.74 (0.55, 0.92)	0.78 (0.58, 0.97)	0.80 (0.45, 1.15)	0.83	1.35 (0.66, 2.78)	0.41
Legumes*⏶ (cups/day)	0.09 (0.05, 0.12)	0.07 (0.03, 0.10)	0.04 (0.02, 0.06)	0.03 (0.00, 0.06)	**0.07**	0.04 (0.00, 1.49)	**0.08**
Micronutrients							
Vitamin A (IU/day)	5428.56 (4404.42, 6452.70)	6957.04 (5234.06, 8680.02)	6394.04 (4846.53, 7941.54)	6933.38 (3951.57, 9915.20)	0.43	1.00 (1.00, 1.00)	0.15
Vitamin D (IU/day)	135.68 (111.55, 159.82)	120.82 (92.61, 149.04)	123.66 (95.44, 151.88)	125.35 (74.52, 176.18)	0.88	1.00 (0.99, 1.00)	0.28
Vitamin E (IU/day)	10.68 (9.26, 12.09)	11.38 (9.40, 13.36)	9.88 (8.20, 11.56)	9.84 (6.86, 12.81)	0.70	1.00 (0.95, 1.05)	0.85
Vitamin K (mcg/day)	95.75 (76.20, 115.30)	122.75 (89.85, 155.64)	93.89 (69.30, 118.48)	115.46 (61.72, 169.20)	0.40	1.00 (1.00, 1.00)	0.27
Thiamin (mg/day)	0.75 (0.70, 0.80)	0.82 (0.75, 0.89)	0.79 (0.73, 0.86)	0.85 (0.72, 0.98)	0.31	1.83 (0.31, 10.65)	0.50
Riboflavin (mg/day)	1.16 (1.07, 1.25)	1.16 (1.04, 1.28)	1.06 (0.96, 1.17)	1.08 (0.89, 1.28)	0.58	0.72 (0.39, 1.35)	0.31
Niacin (mg/day)	13.32 (12.16, 14.49)	13.13 (11.63, 14.64)	12.46 (11.06, 13.86)	12.37 (9.91, 14.84)	0.82	0.99 (0.96, 1.02)	0.40
Pantothenic acid (mg/day)	3.76 (3.31, 4.21)	3.77 (3.18, 4.36)	3.51 (2.97, 4.04)	3.18 (2.32, 4.04)	0.70	0.98 (0.95, 1.02)	0.42
Vitamin B6 (mg/day)	1.21 (1.09, 1.32)	1.19 (1.04, 1.34)	1.14 (1.00, 1.29)	1.05 (0.81, 1.28)	0.72	0.87 (0.65, 1.14)	0.31
Folate (mcg/day)	156.93 (141.84, 172.03)	168.27 (147.03, 189.52)	140.65 (123.29, 158.00)	138.05 (107.79, 168.32)	0.20	1.00 (0.99, 1.00)	0.25
Vitamin B12 (mcg/day)	2.60 (2.24, 2.96)	2.41 (1.97, 2.85)	2.46 (2.02, 2.90)	2.32 (1.59, 3.05)	0.88	0.95 (0.88, 1.04)	0.28
Vitamin C (mg/day)	76.34 (63.73, 88.95)	97.91 (76.68, 119.13)	77.12 (60.78, 93.46)	62.92 (39.23, 86.60)	0.15	1.00 (1.00, 1.00)	0.65
Magnesium (mg/day)	193.15 (179.07, 207.22)	209.79 (189.72, 229.86)	185.13 (167.82, 202.44)	200.95 (167.56, 234.24)	0.29	1.00 (0.99, 1.01)	0.73
Selenium*⏶ (mcg/day)	60.19 (57.35, 63.04)	56.63 (53.11, 60.14)	54.13 (50.84, 57.42)	57.09 (50.93, 63.24)	**0.08**	0.96 (0.93, 0.99)	**0.01**
Iron (mg/day)	7.27 (6.70, 7.83)	7.67 (6.89, 8.46)	7.53 (6.77, 8.28)	9.19 (7.55, 10.83)	0.15	1.11 (0.92, 1.35)	0.28
Copper (mg/day)	0.83 (0.77, 0.89)	0.91 (0.82, 0.99)	0.80 (0.72, 0.87)	0.86 (0.71, 1.00)	0.29	0.96 (0.18, 5.15)	0.96
Zinc (mg/day)	6.14 (5.75, 6.53)	5.84 (5.35, 6.33)	6.04 (5.55, 6.53)	6.21 (5.55, 6.53)	0.79	0.91 (0.74, 1.12)	0.39
Calcium (mg/day)	554.14 (497.82, 610.47)	587.32 (508.95, 665.70)	557.13 (484.48, 629.78)	662.69 (509.13, 816.25)	0.52	1.00 (1.00, 1.00)	0.28

Notes: ^a^Data are presented as mean (95% CI) from linear regression; ^b^data are presented as OR (95% CI) from ordered logistic regression; *omnibus differences *p*-value < 0.10 (bold font); **⏶**OR *p*-value ≤ 0.10 (bold font)

### Model 3 analysis

Data from Model 2 is presented. The analyses from Model 3, which included physical activity as a covariate along with caloric intake, age, race, sex, BMI, and food security, found only two differences from Model 2: (1) a greater average intake of vitamin K (OR = 1.00; 95% CI, 1.00, 1.01; *p* = 0.10) that was associated with having CIPN and (2) no significant association between CIPN severity and tomato (OR = 0.18; 95% CI, 0.01, 2.77;* p* = 0.22) and legume (OR = 0.11; 95% CI, 0.00, 7.28; *p* = 0.30) intake.

## Discussion

The results of this secondary analysis reveal greater intake of refined grains (e.g., including added sugars), but not whole grains, was associated with worse CIPN severity. Our results agree with previous studies that have also found a similar relationship between refined grain intake and CIPN severity [14, 33, 34]. For example, Mongiovi et al. found a marginally significant positive association between citrus fruits and worsened CIPN severity in a sample of survivors of breast cancer (*N* = 1468) [35]. The same study discovered a significant inverse association between grain consumption and CIPN severity when refined and whole grains were combined during analysis. In a sample of survivors of colon cancer (*N* = 74), Compton et al. reported a greater consumption of red and processed meats (*HR* = 1.78) and sugary beverages (*HR* = 1.33) was associated with moderate-severe CIPN, while the risk of severe CIPN was reduced with increased vegetable intake (*HR* = 0.29) [36]. The data suggests diets rich in refined grains and added sugars may have an inflammatory response likely due to the impact on blood glucose and insulin activity that can increase free radical production [37, 38]. The resulting inflammation can harm the peripheral nervous system and act as a potential mechanism for neuropathic pain and worsening CIPN symptoms [5, 39, 40].

Higher fat food groups were consumed in larger amounts by people without CIPN compared to those with CIPN. This is consistent with our prior analysis, which observed those without CIPN ate significantly more total fats (*p* = 0.05), monounsaturated fats (MUFA) (*p* = 0.02), and polyunsaturated fats (PUFA) (*p* = 0.06) compared to those with CIPN [14]. Other studies found that MUFAs and PUFAs have a potential neuroprotective and regenerative role that can reduce oxidative stress placed on peripheral nerves [41]. Foods rich in MUFAs and PUFAs, such as fish, eggs, and nuts/seeds, are also good sources of protein, providing amino acids that support neurotransmitter function [5]. We found a statistically significant inverse association between fish and eggs and having CIPN and worsened CIPN severity. Likewise, greater consumption of nuts and seeds, which are also a good source of MUFAs and PUFAs, resulted in less CIPN severity in this population. The greater dietary intake of MUFAs, PUFAs, and protein from fish, eggs, and nuts/seeds may be protective for peripheral nerves. Although, such findings should be interpreted with caution as the mechanistic relationships between fats, proteins, and peripheral nerve function have not been studied among individuals with CIPN.

Few micronutrients showed significant associations with CIPN. Survivors of cancer with CIPN consumed, on average, greater amounts of thiamine and less selenium compared to those without CIPN. Thiamine is commonly fortified in refined grains in the USA [33], which can explain the greater consumption among participants with CIPN. There is limited research looking solely at the relationship between dietary thiamine intake and nerve function in individuals with CIPN; however, thiamine is involved in neurotransmitter synthesis in the central and peripheral nervous system in general populations [33]. More research is needed to determine the outcomes of excessive thiamine intake. Selenium is a mineral commonly found in fish and eggs. Previous studies observed selenium as an antioxidant that has neuroprotective features that can be beneficial for neuroplasticity in individuals with CIPN [34, 37]. More research is needed to assess its impact on similar populations.

### Strengths and limitations

The cross-sectional study design allowed for the use of multiple validated surveys to demonstrate the relationship between diet and CIPN at one time point. The VioScreen™ Research FFQ and PRO-CTCAE numbness and tingling severity surveys quantified dietary intake and CIPN severity, respectively. Using a larger *p*-value assisted in the detection of potential food groups and micronutrients of interest for further studies to research. However, the limitations of this study are clear. The demographic and clinical characteristics of this study sample were nearly homogeneous with 93% of participants identifying as White, 93% as female, and 63% as survivors of breast cancer. Secondly, there is a lack of certainty about the direction of diet and CIPN associations, meaning that dietary behaviors could be changing the status and severity of CIPN or CIPN status could be altering dietary choices. However, research has shown there is little variation in diet between pre- and post-cancer diagnosis [38]. Third, the timing and self-reported nature of the FFQ can affect dietary intake data. For instance, the seasonality of produce can result in dietary changes depending on the time this study was administered. However, studies have observed minimal seasonal variability in dietary intake from individuals who live in areas with seasonal fluctuation [42]. Additionally, the VioScreen™ FFQ is a self-reported survey that can be susceptible to recall bias. However, the FFQ asked for dietary intake in the last 30 days which is more reliable than other FFQs that ask people to recall intake from the last year [43]. Finally, more research is needed in larger prospective observational and cohort studies with diverse patient populations to investigate the dietary impact on CIPN incidence and severity.

### Clinical implications

Many survivors of cancer are interested in non-pharmaceutical methods, including dietary intake, to manage CIPN-related pain [44]. This study’s results highlighted food groups and micronutrients that may play a role in the augmentation of CIPN severity among post-treatment cancer survivors. The American Institute of Cancer Research (AICR) recommends high-quality dietary patterns to improve health and well-being [45]. Despite the hypothesis-generating nature of this study, registered dietitians can consider recommending AICR diets that are consistent with the findings of this study when approaching patients experiencing CIPN.

## Conclusion

This study exhibits the associations between food group and micronutrient intakes and the development of CIPN in survivors of cancer. A greater intake of lower-quality foods such as refined grains was associated with worsened CIPN severity, while high-quality fat sources and micronutrients from food groups such as eggs and fish were associated with less severe CIPN [14]. Further research is needed to explore dietary interventions focused on increasing high-quality fats and lowering added sugar to prevent or manage CIPN among cancer survivors receiving neurotoxic chemotherapy.

## Data Availability

No datasets were generated or analysed during the current study.
